# Simultaneous biodegradation of λ-cyhalothrin pesticide and *Vicia faba* growth promotion under greenhouse conditions

**DOI:** 10.1186/s13568-022-01383-0

**Published:** 2022-04-15

**Authors:** Aisha A. Abdelkader, Mary S. Khalil, Mahmoud S. M. Mohamed

**Affiliations:** Department of Botany and Microbiology, Faculty of Science, Cairo University, Giza, 12613 Egypt

**Keywords:** λ-cyhalothrin, *Bacillus subtilis*, Pyrethroid, Plant growth promotion

## Abstract

λ-cyhalothrin is a widely used synthetic pyrethroid insecticide and its persistence in plant, soil and water exerts a detrimental effect on humans as well as the environment. There are many studies regarding isolated bacteria capable of degrading λ-cyhalothrin in vitro*.* However, limited work has been done examining the microbial degradation of λ-cyhalothrin together with plant growth promotion under greenhouse conditions. In this study, 43 bacterial strains were isolated from heavily polluted soil with λ-cyhalothrin by the enrichment technique. The plant growth promotion characteristics of all isolates were evaluated. The results revealed that five isolates were potential in λ-cyhalothrin biodegradation at high concentration (1200 mg/L) within only 24 h together with their high plant growth promotion abilities. The morphological, biochemical and 16S rDNA sequence analyses identified the isolates as *Bacillus subtilis* strains. The GC/MS analysis revealed that the selected isolates reached high levels of degradation after only two days, the degradation percentage ranged from 95.72 to 99.52% after 48 h of incubation. Furthermore, the degradation pathway for complete detoxification and metabolism of λ-cyhalothrin was established. Moreover, greenhouse experiment was conducted, the results indicate that the application of seed coat significantly enhanced *Vicia faba* seedling growth and caused an increase from 38.4 to 40.2% percentage of fresh and dry weight, respectively compared to untreated control. All isolates were effective to remove the pesticide residues in *Vicia faba* seedlings and recorded the highest degradation percentage of 83.79 under greenhouse conditions. Therefore, it can be concluded that the *Bacillus subtilis* strains isolated in this study have a dual potential role in complete mineralization of λ-cyhalothrin residues in vivo as well as effective biofertilization for future use in sustainable agriculture.

**Supplementary Information:**

The online version contains supplementary material available at 10.1186/s13568-022-01383-0.

## Introduction

Many countries use a significant amount of agro-chemicals such as fertilizers and pesticides to enhance and preserve the agriproducts supply. Pesticides are thought to be a good solution to secure the ever-increasing worldwide demand of crop yields by combating damage caused by agricultural pests. Recently, the COVID-19 pandemic has exacerbated the worldwide food supply by increasing costs and decreasing income for farmers. The pandemic has made it more difficult to find farm labor and bring crops to the market. Therefore, the application of pesticides has grown enormously by farmers to meet their economic targets (Sarkar et al. 2021).

Globally, approximately 3.5 million tons of pesticides are used annually (Sharma et al. 2019). Although insecticides protect plants and increase the crop production yield, they cause dire environmental pollution problems because of their persistence and other detrimental effects on plants, animals, humans, and soil microorganisms (Alengebawy et al. 2021). It has been estimated that approximately 0.1 percent of pesticides sprayed on farms reach their intended targets; the remainder are spread to ecosystems, contaminating land, water, and air putting lives in jeopardy (Primentel and Levitain 1986). The persistence substances at non-lethal dosages impair the brain system, hepatocytes, lymphocytes, hormone balance, reproduction, embryogenesis, and population increase in fish (Palma et al. 2009; Madrigal et al. 2021).

One major category of insecticides is synthetic pyrethroids (SPs). The natural pyrethrin compounds showed an effective insecticidal activity but were instable in the environment. Modifications in the molecular structure of pyrethrins led to the synthesis of SPs which are more stable and efficient than native pyrethrins in direct sunlight and diverse environmental conditions. These characteristics made them more convenient for use in agriculture (Cycoń et al. 2017). The insecticidal potency of pyrethroids relies on the induction of a toxic effect in the cells of the central and peripheral nervous system of insects (He et al. 2008). Pyrethroids induce insect paralysis then eventual death of insects by permitting a flux of sodium, calcium and chloride ions (Burr and Ray 2004; Hintzen et al. 2009).

In the last few decades, SPs such as fenpropathrin, λ-cyhalothrin, permethrin, cypermethrin, fenvalerate, cyfluthrin, deltamethrin and bifenthrin have been widely used in agriculture, household plants and green landscapes to get over the use of the more toxic and environmentally persistent organochlorine and organophosphorus pesticides (Katsuda 1999). It has been recorded that sales of SPs reach about 1.3–1.4 billion dollars worldwide and constitute nearly 17% of global insecticide sales every year (Zhang 2018). Currently, pyrethroids estimate more than 25% of the world’s total pesticide market (Chen et al. 2011). Because of their overuse, SPs residues have been frequently found in soils, sediments, natural waters and agricultural products (Gu et al. 2008). The main exposure routes of SPs to humans are through skin contact and inhalation (Saillenfait et al. 2015). However, SPs residues were also discovered in solid prepared food, indicating that these substances can also reach humans via food chains (Morgan et al. 2018).

Many types of SPs such as λ-cyhalothrin, fenpropathrin and cypermethrin have been classified as ‘‘moderately hazardous’’ (Class II) by the World Health Organization. Hence, it is crucial to remove SPs residues away from the ecosystem (Wang et al. 2011). Generally, SPs can be degraded through both abiotic pathways, including photo-oxidation and chemical oxidation and biotic pathways mainly by soil microflora (Guo et al. 2009). Microorganisms-induced degradation makes it easier for SPs remediation because it is flexible, cost-effective, and better for the environment (Zhao et al. 2021). Microorganisms characterized by their ability to degrade one or two kinds of pesticides have been isolated and identified (Cycon and Piotrowska-Seget 2016).

Natural-occurring soil bacteria known as plant growth-promoting rhizobacteria (PGPR) colonize the rhizosphere and the roots of plants, resulting in increased plant growth while also providing protection from certain plant pathogens (Van Loon 2007). Rhizobacteria enhance plant growth through a variety of mechanisms, both direct and indirect. Indole acetic acid (IAA) production, gibberellin production, cytokinin production, nitrogen fixation, phosphate solubilization, and the uptake of essential plant nutrients are just a few examples of the direct plant growth-promoting processes (Spaepen et al. 2009). Strains of some bacterial genera have been identified as PGPR such as *Bacillus, Enterobacter, Pseudomonas,* and *Serratia* (Glick, 1995; Hong et al. 2011). Several PGPR-based products are available for sale in many countries, and even more are under development (Choudhary and Johri 2009). Most of these products contain *Bacillus* species, which produce endospores that help to maintain population stability during the formulation and storage processes.

Recently, some studies have focused on the use of PGPR for bioremediation of polluted soil with heavy metals (Jiang et al. 2008), crude oil (Huang et al. 2004) and pesticides (Cycoń et al. 2017). However, scarce data are available about the interactions that may occur as a result of the combined application of PGPR and synthetic pesticides in plant under greenhouse conditions. Therefore, an attempt has been made in the current study in order to find potential PGPR bacterial candidates capable of completely detoxify λ-cyhalothrin not only in lab scale but also under greenhouse conditions. Several bacterial isolates were screened for their capability of enhancing plant growth in addition to efficient degradation of pesticide. Moreover, GC–MS techniques were applied for characterization of the pesticide degradation products of bacterial strains and structural elucidation of the biodegradation mechanisms in vitro as well as detection of the pesticide residual metabolites inside the plant.

## Materials and methods

### Collection of polluted soil samples

The soil samples used in the present study were collected from the soil rhizosphere (10.0 cm depth) of two different sites located in Giza, Egypt with pesticide application history. One site was from cucumber, pepper, onion and cabbage fields at (29.883761745275983°N, 31.261492915228857°E) and the other one was from nectarine and mango fields at (30°07′25.4"N, 31°00′26.5"E). Samples were collected aseptically in sterilized polyethylene bags, labeled (date, time, and quantity) and transported to the laboratory while stored at 4 °C until inoculation.

### Isolation of λ-cyhalothrin degraders bacteria

The enrichment of the bacteria was performed by suspending five grams of soil samples in sterilized 250 mL Erlenmeyer flasks containing 100 mL minimal medium (MM) (Na_2_HPO_4_ 2.0 g/L, KH_2_PO_4_ 0.75 g/L, MgSO_4_.7H_2_O 0.5 g/L, NH_4_Cl 1.0 g/L, and pH 7.5) supplemented with λ-cyhalothrin (10 mg/L) as a sole carbon source. The cultures were incubated at 30 °C in a rotary shaker (150 rpm). Seven days post incubation, aliquots (10%, v/v) were inoculated into another fresh MM supplemented with λ-cyhalothrin (100 mg/L) and incubated for another seven days. After several serial transfers, samples were spread on MM agar plates containing λ-cyhalothrin (100 mg/L). Morphologically different colonies were transferred into sterilized MM agar plates containing increasing concentrations of λ-cyhalothrin (100, 200, 400, 800, 1200 mg/L) as sole carbon and nitrogen source. Bacterial isolates that could grow at the previous concentrations were selected, purified and maintained at 4 °C on Luria Bertani (LB; Laboratories Conda SA, Madrid, Spain) medium as well as 50% glycerol stocks at − 80 °C for longer preservation.

### Determination of auxiliary characteristics of selected bacterial isolates

#### Indole acetic acid (IAA) production by λ-cyhalothrin degrading bacterial isolates

IAA production was performed in LB broth supplemented with 10 g/L filter sterilized L- tryptophan solution (dissolved with aid of sodium hydroxide). For each isolate; 50 mL sterilized LB broth medium were inoculated with 0.1 mL bacterial culture of optical density (OD_600_) 0.5. Inoculated media were incubated at 37 ± 1 °C for 48 h. After incubation, the bacterial cells were collected from the culture broth by centrifugation at 5000 rpm for 15 min. To estimate IAA: the supernatant (2 mL) was mixed with 4 mL of Salkowaski’s reagent (50 mL 35% perchloric acid, 1 mL 0.5 N FeCl_3_ solution and two drops of ortho-phosphoric acid). Development of pink color indicated IAA production. The reading was determined spectrophotometrically at 530 nm and each individual experiment was repeated three times. Using pure IAA (Hi-media, India) as a standard, the concentration of IAA produced by each bacterial culture was estimated using IAA standard curve (Chrastil 1976).

#### Ammonia production by λ-cyhalothrin degrading bacterial isolates

All Isolates were tested for the generation of ammonia in peptone water medium (peptone 10 g/L NaCl, 5 g/L yeast extract, and pH 7.6). Fresh cultures were inoculated into test tubes each containing 10 mL sterilized peptone water and incubated at 37 ± 1 °C for 48–72 h. After incubation, each culture was centrifuged at 4000 rpm for 10 min. Nessler’s reagent (0.5 mL) was added to 10 ml supernatant of each tube. Production of brown to orange color was a positive test for ammonia production. Using pure anhydrous ammonium chloride as a standard, the absorbance was read at 425 nm. The amount of ammonia produced by the culture was calculated using ammonia calibration curve (Cappuccino and Sherman 1992).

#### Hydrogen cyanide (HCN) generation

Hydrogen cyanide production was qualitatively assayed by the method suggested by (Lorck 1948). Bacteria were streaked into sterilized nutrient agar plates amended with glycine (4.4 g/L). Then Petri-plates were inverted and a piece of a Whatman filter paper no.1 impregnated with 0.5% picric acid and 2% sodium carbonate was placed in the lid. Petri-plates were sealed with a parafilm and incubated at 28 °C for 96 h. Discoloration of the filter paper from yellow to orange-brown color indicates HCN production.

#### Siderophore assay

Bacterial strains were screened for siderophore production according to chrome azurol sulphonate (CAS) assay developed by Schwyn and Neilands (Schwyn and Neilands 1987). Quantitative estimation of siderophore production by bacterial strains was done by modified microplate method (Arora and Verma 2017). In brief, 5 µl of freshly grown bacterial culture (10^8^ CFU/mL) were inoculated in 0.5 ml sterilized LB broth in a microcentrifuge tube. After incubation at 37 °C for 48 h, bacterial cultures were centrifuged at 10,000 rpm for 10 min, cell pellets were discarded, and 100 µl of each bacterial supernatant were added in separate wells of polystyrene microtiter plates followed by the addition of 100 µl CAS reagent and were recorded after 20 min absorption at 630 nm using microplate reader (Spectra Max M5e). The experiment was repeated three times for each strain. Siderophore production by strains was measured in percent siderophore unit (psu) which was calculated according to the following formul:

$$\mathrm{Siderophore\, production }(\mathrm{psu}) =\left(\mathrm{Ar}-\mathrm{As}\right)\times \frac{100}{\mathrm{Ar}}$$

where Ar; absorbance of reference (CAS solution and un-inoculated broth), and As; absorbance of sample (CAS solution and cell-free supernatant of sample).

#### Phosphate solubilization assay

In order to test the ability of organisms to solubilize phosphate, fresh isolates were spot inoculated using sterile tooth picks onto Pikovskaya’s Agar medium (Pikovskaya 1948). Plates were incubated for 72 h at 37 ± 1 °C. Formation of a halo zone around the colony indicated a positive result. The diameter of halo zone and that of the bacterial colony were measured and phosphate solubilization index (SI) was calculated by using the formula (Premono et al. 1996): SI (Solubility Index) = colony diameter + halo zone diameter/colony diameter.

### Bacterial identification

#### Morphological and physio- biochemical characteristics

The potent bacterial isolates were selected and identified based on both morphological and biochemical characteristics using Bergey's Manual of Determinative Bacteriology (Bergey 2009).

#### Molecular identification of the most potent bacterial isolates using 16S rDNA sequence analysis

The genomic DNA was extracted from pure colonies of the selected isolates grown overnight on LB medium using Genomic DNA extraction Kit (QIAGEN, Hilden, Germany) according to the kit's instructions. Then PCR was conducted to amplify 16S rDNA of the most potent λ-cyhalothrin PGPR bacterial isolates by the universal primers as described before (Almuhayawi et al. 2021). The obtained PCR amplicons from each isolate were purified and sequenced using the same primers by Macrogen (Seoul, Korea). With the help of DNAStar Lasergene software (V. 7), each isolate's assembled contig sequence was created from forward and reverse sequence reads. The obtained contig sequence of bacteria was compared to references of 16S rDNA gene sequences of other bacterial isolates in GenBank by using National Center for Biotechnology Information (NCBI) BLAST server, and then sequences were automatically aligned using MUSCLE. The phylogenetic relationship for the selected bacterial isolates was constructed using the MEGAX program using the maximum likelihood method and the Kimura 2-paramater model. 1000 bootstrap replications were used to evaluate the data (Edgar 2004; Almuhayawi et al. 2021).

#### Identification of λ-cyhalothrin metabolites produced by bacteria in vitro using gas chromatography/mass spectrometric (GC/MS) analysis

In order to identify λ-cyhalothrin and its metabolites during the bacterial biodegradation, GC/MS technique was employed. In brief, five selected bacterial isolates were inoculated into MM containing 100 mg/L λ-cyhalothrin. The non-inoculated samples containing the same concentration of λ-cyhalothrin were used as control. At appropriate intervals, samples were collected by centrifugation at 14,000 rpm. The supernatant was acidified to pH 2 with 2 N HCl then extracted in equal volume of ethyl acetate solvent. The organic layer was dehydrated, dried and re-dissolved in methanol according to Tallur et al. (Tallur et al. 2008). Extracts were filtered with 0.45 µm membrane (Millipore, Merck, Germany), then analyzed by GC/MS (Agilent, USA). The analysis was performed on a HP-5MS capillary column (30.0 m × 250 µm × 0.25 µm) with an Agilent 6890 N/5975 GC/MS system equipped with automated split/splitless injection system, and with an array of detection ranging from 30 to 500 nm (total scan). The column temperature was held initially at 90 °C for 2 min, and increased to 150 °C at the rate of 6 °C/min for 1 min, then increased to 180 °C at the rate of 10 °C/min for 4 min, and finally increased to 260 °C at the rate of 20 °C/min for 10 min. The ionization energy was 70 eV, and the temperatures corresponding to transfer line and the ion trap were 280 and 230 °C, respectively. The injection volume was 1.0 µL with splitless sampling at 250 °C. The carrier gas (Helium) flow rate was 1.5 ml/min. λ-cyhalothrin and its degradation intermediates identified by mass spectrometry analysis were matched with authentic standard compounds from the National Institute of Standards and Technology (NIST, USA) and WILEY library database as well as with the literatures mass spectral data (Chen et al. 2013, 2014). In order to quantify the residual λ-cyhalothrin after bacterial degradation, analytical standard λ-cyhalothrin from Merck (Darmstadt, Germany) was used for calibration of each sample.

### Greenhouse experiment

#### Study of effect of PGP bacteria on growth parameters of *Vicia faba* seedlings after application of λ-cyhalothrin

The selected bacterial isolates were freshly grown in 250 mL Erlenmeyer flasks containing 100 mL sterilized LB broth then incubated in a rotary shaker at 150 rpm for 48 h at 37 ± 1 °C. The cultures were centrifuged at 6000 rpm for 15 min. The bacterial sediments of each culture were mixed with equal amount of sterilized talc powder, Arabic gum and mixed with sterilized water in a sterilized metal tray to form a homogenous slurry for seed bacterization. *Vicia faba* (broad beans) seeds were kindly obtained from Field Crops Research Institute, ARC, Giza, Egypt. The seeds were surface sterilized with 70% ethyl alcohol for 5 min, 1% sodium hypochlorite for 1 min and rinsed five times with sterile water. A group of sterilized seeds were coated with the resulting slurry from each isolate and left to dry for 24 h under aseptic conditions. Another group of seeds were coated with slurry without bacteria as a control. Plastic pots (20 cm-width) were filled with autoclaved sandy clay soil (1:1 v/v), to eliminate endogenous microbial community, each pot planted with four *Vicia faba* seeds and the pots were kept in the greenhouse at 28 ± 2 °C with a 16 h photoperiod and at 60% humidity. Plants were equally sprayed with λ-cyhalothrin (200 mg/L) at the beginning of dark photoperiod after three weeks of planting; at this age the plants had fully developed true leaves. The experiment was arranged in a randomized block design in five replications and the whole experiment was repeated twice. The plant growth parameters of seedlings were analyzed at the end of the experiment.

#### Quantification of residual λ-cyhalothrin in treated *Vicia faba* seedlings

The collected plant samples after pesticide application were minced using a stainless-steel blender, and representative subsamples of 50 g were homogenized with 150 ml of acetone for 2 min. The resulting macerate was filtered through a clean cotton pad and collected in a measuring cylinder. Afterwards, the extract was supplemented with one-tenth volume of saturated sodium chloride solution and shaken with 200, 100 and 50 ml of methylene chloride successively in a separating funnel. The combined organic phases were filtrated through anhydrous sodium sulphate. Extracts were evaporated till dryness by means of a rotary evaporator operated at 40 °C. The residual extract was dissolved in 10 mL elution solvent (methylene chloride: n-hexane: acetonitrile; 50: 48.5: 1.5) then transferred and processed quantitatively through a 2.5 cm i.d. chromatographic column filled with florisil (7 cm layer) prepared with n-hexane. The residual extract was rinsed several times with ca. 5 mL fractions of the eluting solvent, and each rinse was applied to the column just before the previous fraction had completely entered the column. Elution was resumed until a total of 200 mL of the eluting solvent was applied at a flow rate of about 1.5 mL/min. The eluents were harvested in rotatory evaporator until dryness at 40 °C. For GC analysis, the residue was dissolved in 10 mL ethyl acetate, filtered with 0.45 µm membrane filter. The analytical standard λ-cyhalothrin was dissolved in ethyl acetate to obtain a known concentration of pesticide. Both samples were injected into GC/MS under the same operating conditions as previously mentioned. The retention time of λ-cyhalothrin was recorded at 33.68 min. Quantitation of λ-cyhalothrin in the plant samples was performed by comparing the detector response (area) for the sample to that of the calibration standard (IEEE Engineering in Medicine and Biology Society, 2008).

#### Re-isolation of tested bacteria from *Vicia faba* seedlings

For re-isolation of PGP bacteria from *Vicia faba* seedlings, the leaves, stems and roots were washed with sterilized water to eliminate adhered soil particles and any microorganisms adhered to plant surfaces, then all surfaces were first sterilized by ethyl alcohol 70% for 10 min, then 2.5% sodium hypochlorite mixed with 0.1% tween 20 for 15 min, and rinsed five times with sterile double distilled water. Each plant part was cut into 2 cm long pieces with a sterile blade. Sections were aseptically transferred to sterilized MM agar plates supplemented with 100 mg/L λ-cyhalothrin. Following the incubation time 24 h, the resulting bacterial isolates were identified based on morphological characteristics after purification on different cultivation media and microscopic examinations.

## Results

### Isolation and characterization of λ-cyhalothrin degrading strains

Forty-three morphologically different bacterial isolates have been purified from two agricultural area of polluted soil using enrichment cultures technique. The isolates were purified successfully on MM agar plates supplemented with 100 mg/L λ-cyhalothrin after incubation for 24 h at 37 °C. The isolates were able to grow in MM agar plates supplemented with λ-cyhalothrin at concentrations ranging from 200 up to 400 mg/L at 37 °C. However, when the concentration of λ-cyhalothrin was doubled from 400 mg/L to 800 mg/L, only 13 bacterial isolates were found to be able to grow. It was noticed that some bacterial isolates needed prolonged incubation time to produce visible growth in plates; 48–72 h. At high concentration of 1200 mg/L λ-cyhalothrin, only five isolates designated as 4B, 5A, 7A, 11B and AHB were able to maintain growth on MM agar plates after incubation for 24 h at 37 °C. It was observed that the size of bacterial colonies was reduced by increasing the λ-cyhalothrin concentration in the MM media under the same culture conditions and inoculum size. Furthermore, no growth was observed for all bacterial isolates at concentration 2000 mg/L λ-cyhalothrin in solid MM media after incubation for five days at 37 °C.

### Screening for plant growth promoting λ-cyhalothrin-degrading bacterial isolates

#### Production of indole acetic acid (IAA), ammonia and hydrogen cyanide (HCN)

In order to select potent bacterial isolates, IAA hormone, ammonia and HCN production of λ-cyhalothrin degraders were first tested. The results revealed that most of bacterial strains under investigation produced a significant amount of IAA in the presence of tryptophan (Table 1). Bacterial isolate 5A recorded the highest concentration of IAA 4.75 ± 0.06 µg/mL, whereas the lowest concentration of IAA was 0.07 ± 0.01 µg/mL by bacterial isolate 7A. The isolates C1XB, AHB, 4B, 11B, 7A and 5A were the best IAA producers in vitro with mean IAA concentrations of 3.21, 3.88, 4.07, 4.1, 4.13 and 4.75 µg/ml respectively. In ammonia production test, all the bacterial isolates were producers. The isolate A30 recorded the highest amount 7.88 ± 0.17 mg/L, while isolate A39 produced the least amount of ammonia, 0.84 ± 0.02 mg/L (Table 1). Qualitative measurement of HCN demonstrated that a total of 13 isolates (30.23%) produced high amounts of HCN. The levels of HCN production were moderate and low in 12 isolates and the rest of tested isolates were not able to produce HCN (18 isolates).

**Table 1:** Plant growth-promoting activities of λ-cyhalothrin-degrading strains

Isolate no	IAA concentration (µg/ml)	Ammonia concentration (mg/L)	HCN production	Siderophore production (psu)	Phosphate solubilization
					Solubility index (SI)
A1	0.75 ± 0.03	3.23 ± 0.33	−	–	9.70 ± 0.53
A2	0.23 ± 0.03	2.33 ± 0.5	−	32.54 ± 14.26	10.37 ± 0.64
A3	1.53 ± 0.0	6.4 ± 0.27	−	47.22 ± 8.54	17.45 ± 0.95
A4	0.25 ± 0.02	5.96 ± 0.11	−	8.33 ± 21.08	6.40 ± 0.18
A5	2.12 ± 0.01	2.15 ± 0.17	−	9.46 ± 24.84	1.12 ± 0.02
A6	2.15 ± 0.01	3.04 ± 0.22	+	–	–
A7	0.07 ± 0.01	1.94 ± 0.49	+	11.27 ± 25.87	–
A8	0.12 ± 0.01	4.48 ± 0.29	+	47.22 ± 8.54	–
CIXB	3.21 ± 0.02	5.39 ± 0.05	+	37.32 ± 16.35	9.16 ± 0.27
21	1.24 ± 0.0	5.98 ± 0.14	+	45.72 ± 11.4	9.35 ± 0.26
16	1.72 ± 0.0	2.32 ± 0.19	+	9.73 ± 11.92	1.24 ± 0.02
A12	0.11 ± 0.0	4.76 ± 0.27	+ +	18.55 ± 8.28	20.74 ± 1.28
A13	0.99 ± 0.0	5.38 ± 0.14	+ +	10.40 ± 3.9	8.14 ± 0.24
A14	3.03 ± 0.02	2.53 ± 0.08	+ +	–	–
A15	1.29 ± 0.01	6.93 ± 0.07	+ +	–	19.70 ± 1.22
A16	0.83 ± 0.0	6.51 ± 0.6	+ +	–	4.78 ± 0.19
A17	0.76 ± 0.02	2.63 ± 0.47	+ +	–	–
A18	2.43 ± 0.01	4.88 ± 0.78	+ + +	–	6.53 ± 0.24
A19	1.09 ± 0.01	5.22 ± 0.32	+ + +	54.27 ± 14.2	1.16 ± 0.06
A20	1.71 ± 0.03	1.83 ± 0.18	+ + +	–	–
A21	2.25 ± 0.08	5.81 ± 0.14	+ + +	68.29 ± 8.45	–
A22	1.46 ± 0.06	6.04 ± 0.06	+ + +	72.48 ± 6.16	–
7A*	4.13 ± 0.06	3.43 ± 0.11	+ + +	83.14 ± 6.51	3.58 ± 0.04
5A*	4.75 ± 0.06	3.24 ± 0.14	+ + +	82.48 ± 1.58	5.39 ± 0.11
C1*	1.96 ± 0.06	6.37 ± 0.06	+ + +	75.53 ± 3.66	6.11 ± 0.18
4B*	4.07 ± 0.06	5.64 ± 0.11	+ + +	72.68 ± 2.38	3.04 ± 0.08
AHB*	3.88 ± 0.05	6.25 ± 0.11	+ + +	89.31 ± 3.57	4.03 ± 0.06
A28	2.06 ± 0.05	5.96 ± 0.15	+ + +	71.29 ± 3.44	–
A29	1.46 ± 0.05	3.44 ± 0.08	+ + +	–	–
A30	2.33 ± 0.08	7.88 ± 0.17	−	68.97 ± 7.21	–
A31	0.34 ± 0.02	5.94 ± 0.1	−	–	–
A32	2.83 ± 0.03	2.19 ± 0.13	−	78.12 ± 4.84	–
A33	1.97 ± 0.03	3.18 ± 0.05	−	–	–
A34	0.15 ± 0.02	2.36 ± 0.1	−	–	–
A35	0.13 ± 0.01	4.22 ± 0.15	−	–	–
A36	2.42 ± 0.02	5.19 ± 0.13	−	–	–
A37	1.11 ± 0.06	4.03 ± 0.1	−	37.12 ± 11.51	–
A38	0.2 ± 0.04	1.43 ± 0.07	−	–	–
A39	0.33 ± 0.03	0.84 ± 0.02	−	–	–
A40	0.52 ± 0.01	0.92 ± 0.03	−	–	–
A41	2.27 ± 0.09	6.17 ± 0.11	−	66.25 ± 3.02	1.64 ± 0.05
A42	2.18 ± 0.11	4.50 ± 0.13	+ + +	72.08 ± 2.84	–
11B*	4.10 ± 0.11	3.46 ± 0.1	−	73.87 ± 6.57	2.27 ± 0.03

*IAA* Indole acetic acid, *psu* percent siderophore unit. The values indicate the mean of three replicates. +  +  +  = large amount of HCN; +  +  = moderate amount of HCN; +  = low amount of HCN;−= no HCN production. The “*”Indicates the bacterial isolates that were further characterized in this study

#### Siderophore assay

The production of siderophores by bacterial isolates was assessed using the modified microplate technique (Arora and Verma 2017). Out of 43 bacterial isolates, 26 isolates (60.46%) were observed to produce siderophores, but 17 isolates could not. The amount of siderophore produced ranged from 8.33 to 89.31 psu. The maximum siderophores production was recorded for the isolate AHB followed by 7A and 5A (Table 1).

#### Phosphate solubilization

To evaluate the phosphate solubilization of λ-cyhalothrin degraders, Pikovskaya's Agar medium was used. The results revealed that three isolates (A12, A15 and A3) demonstrated a remarkably high solubility index (SI) recorded 20, 19 and 18 respectively. However, 22 isolates (51.16%) failed to solubilize phosphate.

#### Selection, identification and characterization of the best λ-cyhalothrin degrader

The prospective bacterial isolates were selected based on their levels of λ-cyhalothrin biodegradation at high concentrations (800 to 1200 mg/L) together with their plant growth promotion potentiality (Table 1). Eight isolates designated as CIXB, 21, 7A, 5A, C1, 4B, AHB and 11B were first selected as the most potent ones. Obviously, the morphological colonies characteristics of the eight isolates on LB and MM medium showed that each isolate has colonies that are different in size, shape, margin, elevation and consistency. The Gram- staining and microscopic examination revealed that five isolates (7A, 5A, 4B, AHB and 11B) are Gram-positive rods, some of which are in pairs or chains and endospore formers. Initial identification of these isolates revealed that they belong to the phylum Firmicutes and genus *Bacillus*. Further biochemical tests of the isolates demonstrated that all the strains are motile, catalase-positive, can grow at 6.5% NaCl, can hydrolyze starch and casein, can utilize citrate and glycerol, and are VP (Voges-Proskauer) positive, cannot grow at 55 °C among other characteristics. The biochemical features suggested that the isolates are different strains of *Bacillus subtilis*. On the other hand, the three isolates (CIXB, 21 and C1) are Gram-negative rods coccobacilli which produce pink colonies on MacConkey agar medium. They tested positive for lactose fermentation, catalase and urease production, VP, methyl red and citrate utilization within 48 h. In addition to this, they were negative in tests for indole and oxidase production. They were initially identified as members of *Enterobacter* species.

#### Molecular identification and phylogenetic analysis of the potent isolates

**Fig. 1:**
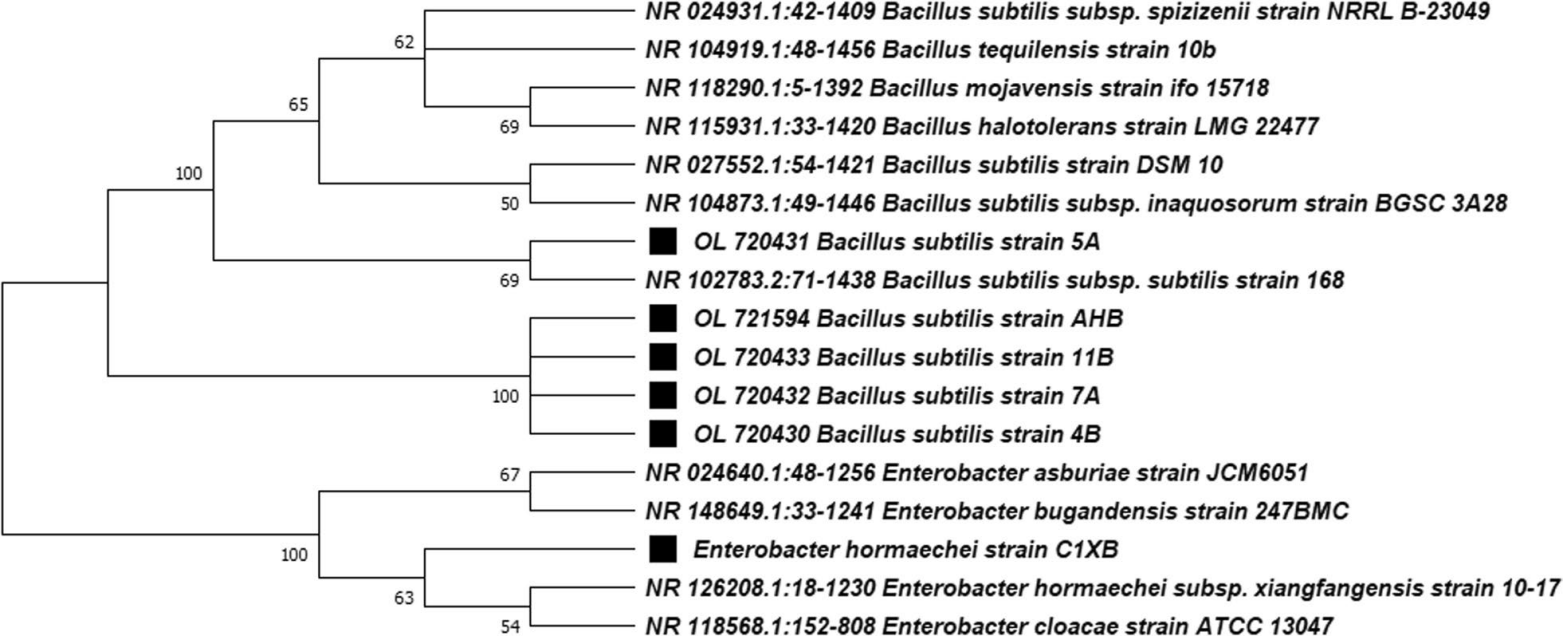
The evolutionary analysis of the best biologically active bacterial isolates as analyzed by phylogenetic tree constructed by the maximum likelihood method using MEGAX software for the 16S rDNA sequences of *Bacillus subtilis* strains 7A, 5A, 4B, AHB and 11B as well as *Enterobacter hormaechei* strain CIXB. The numbers at nodes represent the percentage values given by 1000 bootstrap samples analysis

The best PGP active and likely safe (non-pathogenic), namely 7A, 5A, 4B, AHB and 11B were further analysis to confirm the identity by sequencing the 16S rDNA genes using the universal primers 27F and 1492R. The five isolates were belonged to the genus *Bacillus*, which confirmed the previous morphological and biochemical characterization. The isolates amplicons exhibited a high degree of sequence similarities ranging from 99–100% with 16S rDNA from other *Bacillus subtilis* references strains in GenBank whereas the bacterial isolate CIXB was identified as *Enterobacter hormaechei* that was highly affiliated to *Enterobacter bugandensis* 247 BMC (Fig. 1). All isolate sequences were deposited into the GenBank database under the accession numbers OL720432, OL720431, OL720430, OL721594 and OL720433 for the isolates 7A, 5A, 4B, AHB and 11B respectively. The program MEGAX was used to generate the phylogenetic tree with the related *Bacillus* species using the maximum likelihood method by bootstrap analyses based on 1000 analysis (Fig. 1). The phylogenetic tree separated the selected isolates into two different main groups, *Bacillus* group and *Enterobacter* group. *Bacillus subtilis* 5A in one subgroup of *Bacillus* group, while the other four *Bacillus subtilis* strains 7A, 4B, AHB and 11B were grouped separated in sister phylogenetic subgroup in the same *Bacillus* group (Fig. 1). The bacterial strains were deposited and available in Culture Collection Ain Shams University (Cairo-Egypt), under the numbers CCASU-2022-1 to CCASU-2022-5 for the *Bacillus subtilis* isolates 5A, 7A, 4B, AHB and 11B, respectively.

#### Quantification of residual λ-cyhalothrin for the selected strains

In order to determine the efficiency of λ-cyhalothrin degradation, the amounts of residual λ-cyhalothrin present in each of the selected bacterial culture were analyzed by GC/MS analysis. The λ-cyhalothrin concentrations were drastically reduced only after 24 h of incubation in all tested isolates with initial 100 mg/L λ-cyhalothrin concentration (Table 2). The degradation percentages were calculated and the highest one was recorded for isolate 4B whereas, isolate 7A recorded the lowest degradation percentage 73.24 among the tested isolates. Further incubation for two days increased the λ-cyhalothrin degradation and the isolates 4B and 5A almost degraded the pesticide completely. Interestingly, the other bacterial isolates also recorded high rates of degradation with minimum degradation percentage 95.72 for isolate 7A. The control, non-inoculated medium mixed with 100 mg/L λ-cyhalothrin, showed no evident change after one day of incubation and only 6.71 ± 0.13 percentage of reduction in the λ-cyhalothrin concentration after two days of incubation under the same conditions.

**Table 2:** The percentages of λ-cyhalothrin degradation in MM after incubation at 37 °C for two days

Isolates	Degradation of λ -cyhalothrin (%)	
	24 h	48 h
4B	91.27 ± 0.30	99.52 ± 0.02
5A	88.54 ± 0.40	98.45 ± 0.05
7A	73.24 ± 0.93	95.72 ± 0.15
AHB	78.71 ± 0.74	96.04 ± 0.14
11B	78.34 ± 0.75	95.99 ± 0.14

#### Chemical characterization of λ-cyhalothrin residues produced by the selected bacterial strains

The GC/MS analysis was employed in order to identify the λ-cyhalothrin residues produced by each of the selected isolates during the degradation in MM. Different incubation time points, 24 and 48 h, were tested and the analysis revealed that after 24 h of incubation at 37 °C, it was possible to determine the metabolites of λ-cyhalothrin. The spectrum patterns of λ-cyhalothrin degrading residues produced by the bacterial isolates revealed that a significant peak was detected in all the tested samples after 24 h, with molecular weight (m/z) of 449 and a retention time (RT) of 34.39 min, which was exactly the same as the authentic standard of λ-cyhalothrin in the library databases and the control sample of pure compound. The compound has therefore been identified as λ-cyhalothrin (Additional file 1: Fig. S1). In addition, the intermediate concentration was below the parent λ-cyhalothrin. The peak area of λ-cyhalothrin in the GC/MS spectra of all bacterial cultures was lower than that of the GC/MS spectrum of the uninoculated control. Furthermore, new metabolites were formed in the bacterial cultures, such as bis (4-phenoxyphenyl) methanone, 2-hydroxy-2-(3-phenoxyphenyl) acetonitrile, 2-(3-phenoxyphenyl) acetonitrile, 2,4-Bis(1,1-dimethylethyl)- Phenol, 3-phenoxy-benzaldehyde, Isopropyl 4-hydroxybenzoate, 1-Phenoxy-2-propanol, and phenol (Additional file 1: Fig. S1, Table 3). These compounds were analyzed and identified according to the similarity of their retention times, molecular weights, and molecular formulas to those of the corresponding authentic compounds in the library databases.

**Table 3:** A summary of the metabolites detected by GC/MS during the biodegradation of λ-cyhalothrin by the bacterial isolates after 24 h

No	RT (min)	m/z	Compound	Isolate(s)
1	34.39–34.51	449		4B, 5A,7A, 11B, AHB
2	32.17–32.18	366		5A, 7A, 11B
3	23.97	332		4B
4	20.56	225		4B, 7A
5	24.53–24.54	209		4B, 5A, 7A, 11B, AHB
6	17.05–17.06	206		4B, 5A, 7A, 11B, AHB
7	20.50- 20.51	198		5A, 11B, AHB
8	3.35	180		AHB
9	3.09	152		11B
10	3.22–3.48	94		4B, 5A, 7A

**Fig. 2:**
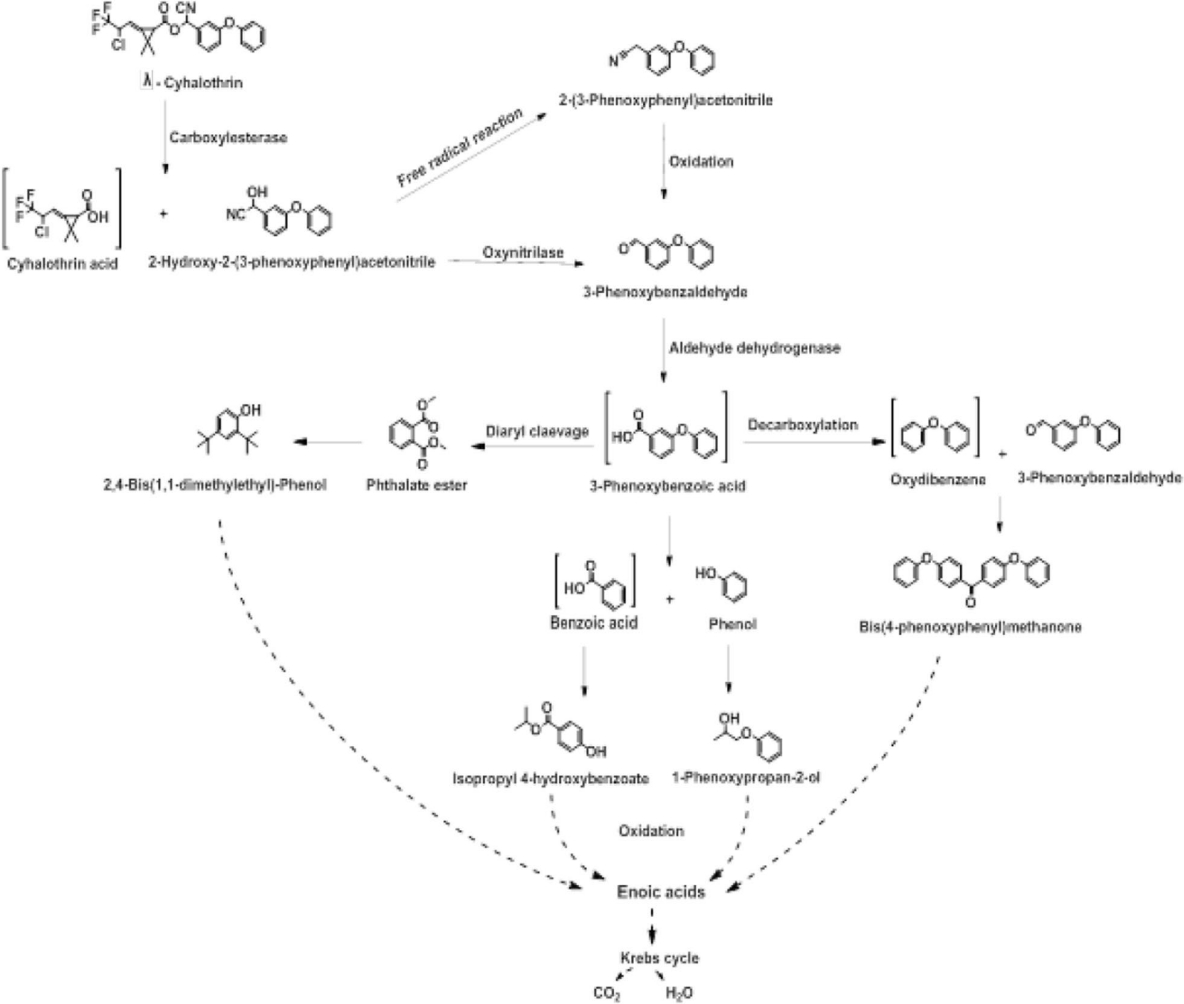
Proposed λ-cyhalothrin biodegradation mechanism in *Bacillus subtilis* isolates based on the detected metabolites

The retention times, molecular mass and chemical structures of the compounds detected by each of the five tested bacterial isolates were summarized in Table 3. It was observed that two different peaks corresponding to λ-cyhalothrin intermediate metabolites at retention times 17.05, and 24.53 were detected in all tested bacteria together with the peak characteristic for to λ-cyhalothrin at 34.39. Furthermore, other three peaks were detected in at least three bacterial isolates, one peak in two isolates and three peaks in only one isolate (Additional file 1: Fig. S1; Table 3). All the detected metabolites were analyzed by comparing the obtained data with the WILEYREGISTRY 8E, MAINLIB, NIST_MSMS and REPLIB libraries as well as the mass spectral data with those reported in literature. Finally, a catabolic degradation pathway for λ-cyhalothrin has been postulated based on the λ-cyhalothrin identified intermediate compounds that have been detected (Fig. 2).

### Greenhouse experiment

#### Effect of λ-cyhalothrin degrading bacteria on broad beans (*Vicia faba*) growth

**Fig. 3:**
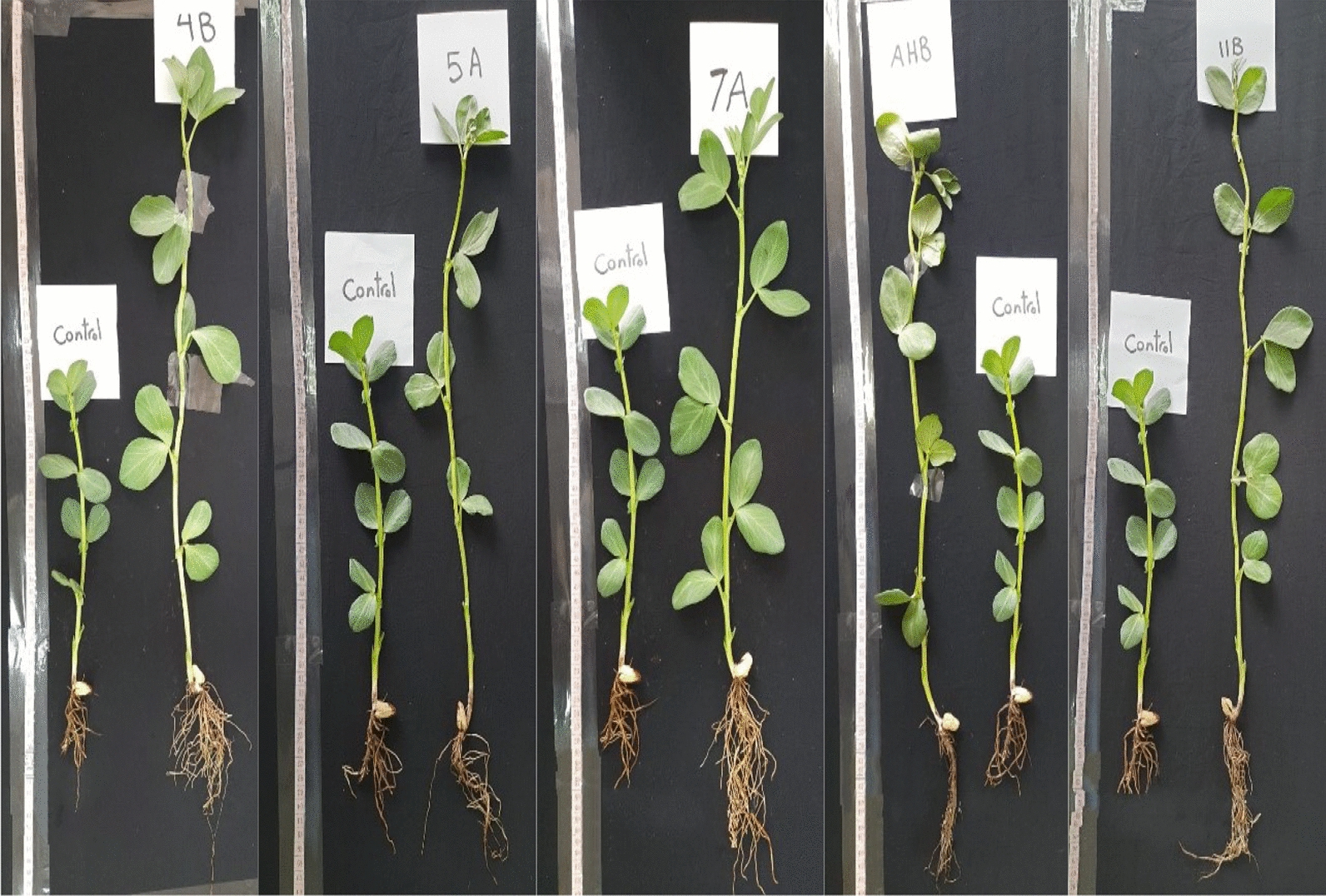
Comparison between growth of treated seedlings and control after 28 days of regular irrigation in pot experiment

**Fig. 4:**
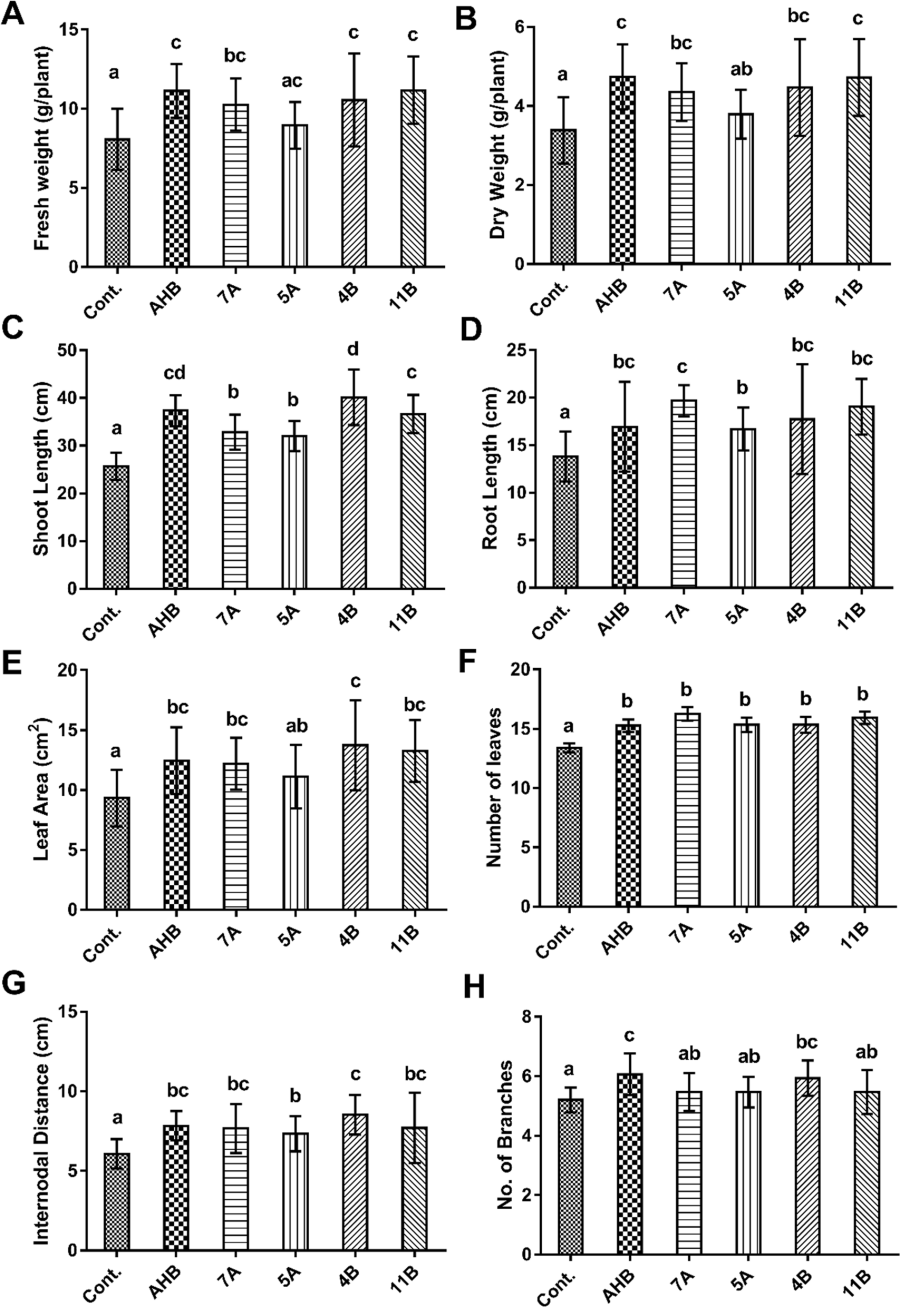
Effect of bacterial treatments on growth of *Vicia faba* seedlings in pot experiment after 4 weeks compared to control (untreated). **A** seedling fresh weight; **B** dry weight; **C** shoot length; **D** Root length; **E** leaf area; **F** number of leaves; **G** internode distance; **H** number of branches. Duncan's tests were used to analyze the data and the results are represented as the mean ± standard deviation. The presence of different letters on the bars indicates a significant difference between strains, whereas the presence of a common letter indicates that the strains are not significantly different (*p* < 0.05)

After four weeks of regular irrigation, the growth parameters of broad bean seedlings were measured and analyzed statistically. The broad beans seed coat with λ-cyhalothrin degrading bacteria caused marked increase in seedling growth parameters in all tested bacteria after λ-cyhalothrin was sprayed (Fig. 3). The increase in fresh weight (FW) and dry weight (DW) was significant and ranged from 10.9–38.4 and 11.7–40.2 percentage, respectively compared with untreated control seedling (untreated seeds). The maximum increase in fresh and dry weight was recorded for the isolates AHB and 11B (Fig. 4A, B). Furthermore, the shoot length, root length and leaf area were the most obvious features that differed significantly between the treatments and control. Isolate 4B exhibited the highest significant increase in shoot length and leaf area recorded 56.36 and 47.14 percentage respectively as compared to the control, whereas isolate 7A recorded the longest roots 42.51 percentage longer than the untreated control (Fig. 4C–E). The numbers of leaves and internodal distance of seedling in all treatment were significantly different from those of the control (Fig. 4F, G). The treatment with isolate 7A and 4B recorded the highest leaves area and internodal distance with an increase of 21.3 and 40.7 percentage, respectively compared with untreated control. However, the number of branches were significantly different from control in treatment with AHB and 4B but not in 7A, 5A and 11B (Fig. 4H).

#### Estimation of residual concentration of λ-cyhalothrin in *Vicia faba* seedlings

GC/MS was employed at the end of greenhouse experiment to determine the resi-dues of λ-cyhalothrin remaining on *Vicia faba* seedling (Table 4). The amount of λ-cyhalothrin in plant extracts was quantified and the results revealed that isolate 5A reduced greatly the residual λ-cyhalothrin concentration and recorded the highest degradation percentage followed by isolate 4B recorded 83.79 and 81.51 respectively.

**Table 4:** Residual λ-cyhalothrin concentration and percentages of degradation in *Vicia faba* seedlings after one week of spraying

Isolate	Residual λ –cyhalothrin (µg/g FW)	Degradation of λ –cyhalothrin (%)
4B	0.351 ± 0.03	81.51 ± 1.42
5A	0.315 ± 0.10	83.79 ± 2.69
7A	0.569 ± 0.04	69.57 ± 6.84
AHB	0.597 ± 0.09	68.77 ± 0.49
11B	1.202 ± 0.15	36.62 ± 7.87
Control	1.914 ± 0.33	–

#### Colonization of tested bacteria in *Vicia faba* seedlings

To investigate the ability of λ-cyhalothrin degrading bacteria to colonize inside the *Vicia faba* seedlings, each part of the seedlings (root, stem and leaves) was aseptically transferred to MM agar plates containing 100 mg/L λ-cyhalothrin. The results demonstrated that bacterial growth was observed around the leaves sections of all treatments. However, no growth was observed in the plates incubated with untreated control seedling plant. Moreover, visible bacterial growth was detected in only the MM plates incubated with stems sections of the treated seedling with bacterial isolates 4B and 5A, but no growth was observed in all plates incubated with roots sections. Further purification and characterization of this bacterial growth indicated similarity to the bacterial isolates that were used in the pot experiment.

## Discussion

The extensive use of neurotoxic pyrethroid pesticides including λ-cyhalothrin in farming practices, to control the insect pests’ losses, execrated the environmental problems due to their accumulation in soil, ground water and food chain (Zhan et al. 2020). In order to restore the environment from the accumulation of this toxic pollutant, bioremediation emerges as an effective, easy and cheap detoxification approach. The bacterial based biodegradation of pyrethroid using plant beneficial bacteria is a new promising area of research and a valuable eco-friendly strategy (Akbar and Sultan 2016; Yadav et al. 2020). However, the stability and efficiency of pesticide biodegrading bacteria along with plant growth promoting potentiality in real environmental conditions remains not studied as frequently as in vitro.

The isolation of λ-cyhalothrin degrading bacteria from agriculture area previously sprayed with λ-cyhalothrin was effective to obtain 43 bacterial isolates by the enrichment technique. Some studies reported the isolation of microorganisms capable of degrading λ-cyhalothrin with the same technique from contaminated sites and agricultural fields (Akbar et al. 2015; Chumro et al. 2017; Zhang et al. 2019). It was reported that initial concentration of λ-cyhalothrin in the cultural media increase the lag phase of the pesticide degradation thereby decrease bacterial growth rate (Jilani and Altaf Khan 2006; Tian et al. 2018; Hu et al. 2019). Screening experiment of all bacterial isolates demonstrated that only five isolates can grow and utilize λ-cyhalothrin efficiently at high concentration (1200 mg/L) within only 24 h without lag phase indicating high efficiency of the selected isolates. This suggests that there is λ-cyhalothrin toxicity on bacteria, and a basal level of resistance provided by each bacterial isolate. In literature, the highest concentration reported for microorganism, that can utilize cyhalothrin as the carbon and nitrogen source, was 800 mg/L for the *Bacillus thuringiensis* strain ZS-19 within only one day (Chen et al. 2015). However, other studies reported prolonged incubation time for the biodegradation process of λ-cyhalothrin (Tian et al. 2018; Palmer-Brown et al. 2019).

In order to reduce the usage of chemical fertilizers and bioremediate the pesticide pollution, all λ-cyhalothrin degrading bacterial isolates were tested for their ability to enhance plant growth. It is well known that the bacterial production of IAA, phosphate solubilization activity and ammonia production positively affect the physiology of plants through stimulation of root development, affecting photosynthesis, formation of pigments, and biosynthesis of different metabolites (Spaepen and Vanderleyden 2011). Whereas, the siderophores and hydrogen cyanide production indirectly promote plant growth through enhancing antagonism to phytopathogens by chelating of metals ions and decreasing the availability of phosphate (Spaepen et al. 2009; Singh et al. 2019).

Interestingly, we found that the best λ-cyhalothrin degrading bacteria (4B, 5A, 7A, AHB and 11B) produced the highest concentration of IAA with maximum production of 4.75 ± 0.06 µg/mL (Table 1), similarly siderophores compared to other isolate, these isolates were also able to produce high amount of HCN, whereas moderate phosphate solubilization activities were recorded. This is in line with the results demonstrated in previous laboratory studies of bioremediation potential combined with plant growth promoting characteristics that have been carried out for different pollutants such as chromate (Wani et al. 2007), cypermethrin pesticide (Akbar et al. 2015) as well as fungicide (Myresiotis et al. 2012).

Based on the isolates plant growth promotion results together with the ability of isolates to grow on MM agar plates supplemented with at least 800 mg/L, three isolates were selected for further analysis (CIXB, 21 and C1) beside the five best isolates of λ-cyhalothrin degradation. The identification of these isolates revealed that the Gram-negative isolates belonged to *Enterobacter* genus and due to their potential pathogenicity, they were excluded. On the other hands, the Gram-positive isolates belonged to *Bacillus* genus and were analyzed further. Indeed, the use of sporulating Gram-positive *Bacillus* species in bioremediation, biological control, as a biofertilizer and a biological solution for environmental protection, has an advantage over other bacterial species because it can confer high population stability during formulation and application in the natural environmental matrix (Kokalis-Burelle et al. 2006; Singh et al. 2019; Mandree et al. 2021).

The degradation percentage of λ-cyhalothrin by the selected *Bacillus* isolates were calculated to be ranging from 73.24–91.27% and 95.72–99.52% after 24 and 48 h respectively. These results are drastically elevated as compared to the reported λ-cyhalothrin degradation percentage 61% and 88% for *Bacillus thuringiensis* strain ZS-19 after 24 and 48 h respectively (Chen et al. 2015) and 69.6% for *Acinetobacter baumannii* ZH-14 after 72 h (Zhan et al. 2018). Moreover, *Bacillus subtilis* BSF01 demonstrated λ-cyhalothrin degradation with percentage 30 and 40% after 24 and 48 h, respectively and the maximum degradation 76.8% after 7 days (Xiao et al. 2015). Other bacteria such as *Brevibacillus parabrevis* FCm9 demonstrated a lower degradation activity against λ-cyhalothrin utilizing only 60.23% within 7 days (Akbar et al. 2015). Taken together, the high degradation efficiency of our isolated bacteria compared to other studies drew a conclusion that these isolates may be ideal for bioaugmentation of the λ-cyhalothrin-contaminated plant and environments.

λ-cyhalothrin belongs to SPs type II that has a cyano group in their structure, which is more toxic and persistent to microbial degradation (Cycon and Piotrowska-Seget 2016). Therefore, further analysis of the λ-cyhalothrin biodegradation pathway was carried out because more toxic metabolites may be produced from the biodegradation of the SPs insecticides (Tyler et al. 2000; Laffin et al. 2010).

The results of identified metabolites indicated that λ-cyhalothrin ester bond was primarily hydrolyzed by carboxylesterases yielding acid and alcohol moieties in all tested isolates which is confirmed by the detection of the intermediate compounds 2-hydroxy-2-(3-phenoxyphenyl) acetonitrile and 2-(3-phenoxyphenyl) acetonitrile (Fig. 2 Table 3). Many species of bacteria reported the same primary hydrolysis of ester bond not only for λ-cyhalothrin but also for other pyrethroids (Chen et al. 2014; Cycon and Piotrowska-Seget 2016; Zhan et al. 2020). The compound 2-hydroxy-2-(3-phenoxyphenyl) acetonitrile was detected in only two isolates indicating its instability and spontaneous transformation to 2-(3-phenoxyphenyl) acetonitrile and 3-phenoxy-benzaldehyde, which were detected in all isolates (Pankaj et al. 2016). Oxinitrilase, an enzyme that converts cyanohydrins to aldehydes, can convert 2-hydroxy-2-(3-phenoxyphenyl) acetonitrile to 3-phenoxybenzaldehyde. In addition, free radical reactions can convert 2-hydroxy-2-(3-phenoxyphenyl) acetonitrile to 2-(3-phenoxyphenyl) acetonitrile. In this regards, radical reactions are ubiquitous in bacteria, and the intermediate of this compound could be stabilized by the cyano and aromatic groups of the substrate (Veum et al. 2006).

The strong antimicrobial activities of 3-phenoxy-benzaldehyde prevent some bacterial species from further degradation of pyrethroid and thereby decrease the efficiency of biodegradation (Tyler et al. 2000). Remarkably, all isolates were able to further degrade 3-phenoxy-benzaldehyde to 1-phenoxy-2-propanol, and isopropyl 4-hydroxybenzoate by aromatic ring cleavage as well as phenol by diphenyl ether cleavage and subsequent catabolism (Cycon and Piotrowska-Seget 2016; Hu et al. 2019; Zhao et al. 2021). The production of phenol and phenyl ethanoic acid, benzoic acid from 3-phenoxy-benzaldehyde has been observed in the two previous cyhalothrin biodegradation studies by *B. thuringiensis* (Chen et al. 2015) and by bacterial consortium of *Bacillus* species, *Bacillus* sp. CBMAI 2065, *Bacillus* sp. CBMAI 2066 and *Bacillus* sp. 2B (Birolli et al. 2019).

In this study, among these identified intermediate metabolites some compounds were reported for the first time in the biodegradation pathway of λ-cyhalothrin such as bis (4-phenoxyphenyl) methanone that may be formed from the decarboxylation of 3- phenoxybenzoic acid to yield diphenyl ether or oxydibenzene that then reacts with 3-phenoxybenzaldehyde (Veum et al. 2006).

All these intermediate products disappeared after prolonged incubation. Detection of high amounts of aliphatic acids such as oleic acid, erucic acid and other enoic acids confirmed complete degradation of λ-cyhalothrin by the bacterial isolates. Bacteria employ the pyrethroid degradation to generate energy by Krebs cycle (Zhao et al. 2021). These results were in line with the high efficiency of all tested isolates in the λ-cyhalothrin biodegradation compared to other studies in literature.

To evaluate the ability of these isolates to detoxify the pesticide combined with plant growth promotion activities, greenhouse experiment was conducted because the tests based on in vitro experiments do not always correlate with activities under natural conditions. The results indicate that the application of seed coat significantly (*p* < 0.05) enhanced *Vicia faba* seedling growth (Fig. 4). Plants treated by seed coat with bacteria were improved and caused an increase from 38.4 to 40.2% percentage of fresh and dry weight, respectively compared to untreated control. The enhancement of *Vicia faba* seedlings growth treated with bacteria is attributed to the substantial IAA, ammonia production and phosphate solubilization by λ-cyhalothrin degrading iso-lates. Many studies reported the beneficial effect of diverse PGPR bacteria on plant growth in normal environment or under biotic or abiotic stresses (Van Loon, 2007; Warrad et al. 2020). The multiple roles of bacteria for plant growth promotion and bioremediation attract many scientists. The phosphate solubilizing bacteria *Pseudomonas* spp. were able to stimulate crop productivity of wheat in the presence of a combination of chlorpyrifos and pyriproxyfen pesticides, however under pesticide stress the effect of bacteria on plant growth criteria was less pronounced (Munir et al. 2019). Similarly, the chlorpyrifos-degrading *Achromobacter xylosoxidans* (JCp4) and *Ochrobactrum* sp. displayed substantial plant growth promoting properties in the presence and absence of the pesticide and were able to bioremediate soil with the chlorpyrifos pesticide (Akbar and Sultan 2016). Furthermore, it was reported that *Bacillus cereus* strain Y1 improved the removal of deltamethrin in Chinese cabbages grown in both greenhouses and open fields with a reduction of 9.1 and 24.4%, respectively compared to controls. In our study, beside the PGP activities all *Bacillus subtilis* isolates were effective to remove the pesticide residues in *Vicia faba* seedlings (Table 4). Although all isolates were identified as *Bacillus subtilis*, the percentage of residual λ-cyhalothrin removal was different among the five strains, this may be related to the ability of each bacterial isolate to survive in the soil as well as inside the plant (Zhang et al. 2016).

Interestingly, we are successfully able to re-isolate the λ-cyhalothrin degrading bacteria from inside the seedling suggesting the ability of bacteria to colonize the intercellular and/or intracellular spaces of host plant, without any plant morphological changes. The effect of plant surrounding environmental conditions either abiotic or biotic factors such as pesticide application, phytopathogen and plant–microbe interactions plays an important role on the structure of endophytic community (Ryan et al. 2008).

The tested λ-cyhalothrin degrading endophytic bacteria are adapted to living inside the *Vicia faba* seedling stem and leaves but not in root suggesting that the endophytic bacteria association were likely enriched in the conducted pesticide treatments. In this regards, The interaction between endophytic bacteria and plants is a complex phenomenon, it was observed that the rhizobia endophytic colonization of rice migrated into the stem base and leaves after root entry indicating the dynamic association between plant and endophytes (Chi et al. 2005).

In conclusion, in this study five potential *Bacillus subtilis* strains have been isolated and characterized for their capabilities to promote the plant growth as well as completely detoxify λ-cyhalothrin as the sole carbon source for growth at high concentrations within only 48 h as analyzed by GC/MS. The isolates were efficient in both roles not only in the laboratory scale but also under greenhouse conditions when they formulated in the seed of *Vicia faba*. Furthermore, we proposed the metabolic pathway for complete degradation of λ-cyhalothrin by all isolates. These *Bacillus subtilis* isolates might be useful in formulating new bioinoculants in agriculture with combinations of biofertilization and bioremediation, leading to a sustainable agricultural production.

## Supplementary Information

**Additional file 1:** **Figure S1.** GC/MS profiles of MM supplemented with λ-Cyhalothrin after incubation for 24 h at 150 rpm and 37 °C.

## Data Availability

Not applicable.

## Abbreviations

SPs: Synthetic pyrethroids
PGPR: Plant growth-promoting rhizobacteria
IAA: Indole acetic acid
MM: Minimal media
GC–MS: Gas Chromatography/Mass Spectrometry
