# Supplementary material for: Simultaneous biodegradation of λ-cyhalothrin pesticide and Vicia faba growth promotion under greenhouse conditions
Source: AMB Express. 2022 Apr 15;12:44. doi: 10.1186/s13568-022-01383-0 (PMC9012055; doi:10.1186/s13568-022-01383-0)
Supplement: Supplementary file 1 — Additional file 1: Figure S1. GC/MS profiles of MM supplemented with λ-Cyhalothrin after incubation for 24 h at 150 rpm and 37 °C. [file 13568_2022_1383_MOESM1_ESM.pdf]

Supplementary Figures

A

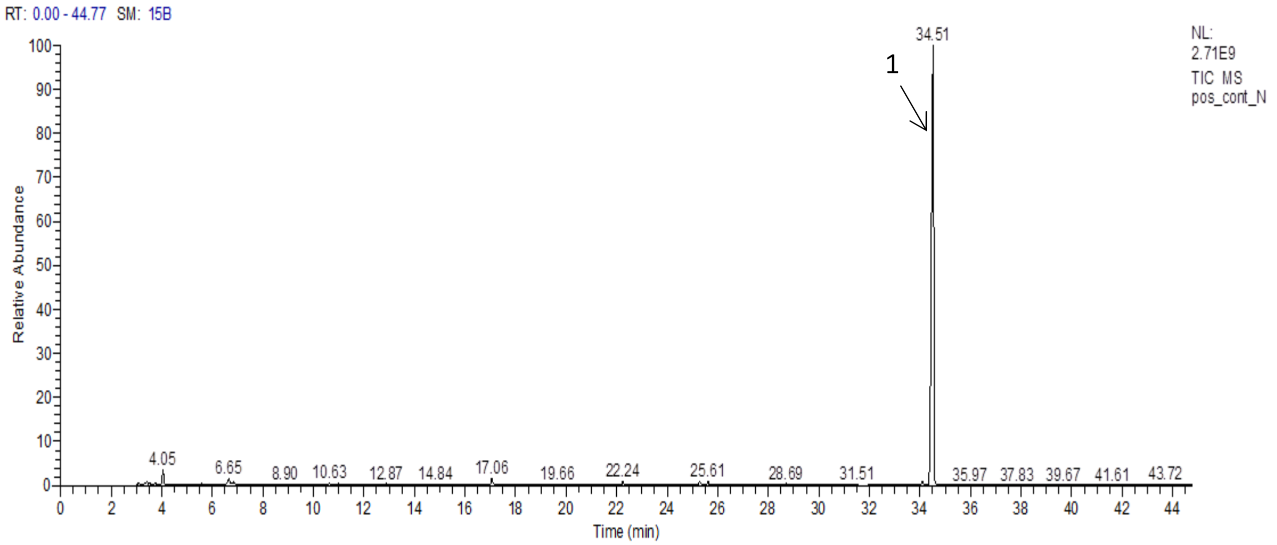

B

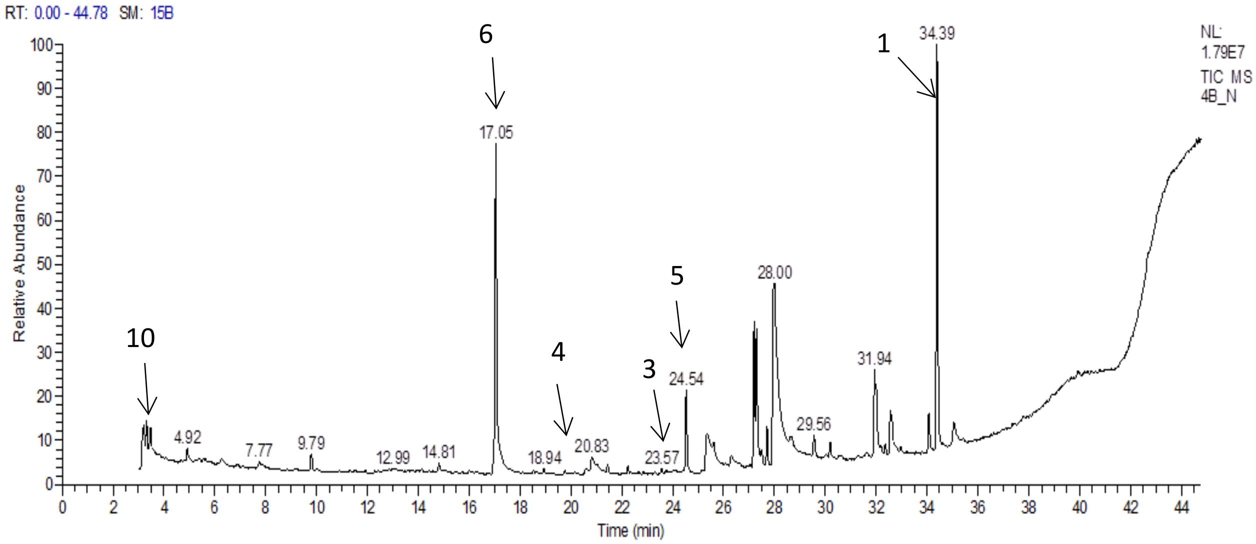

C

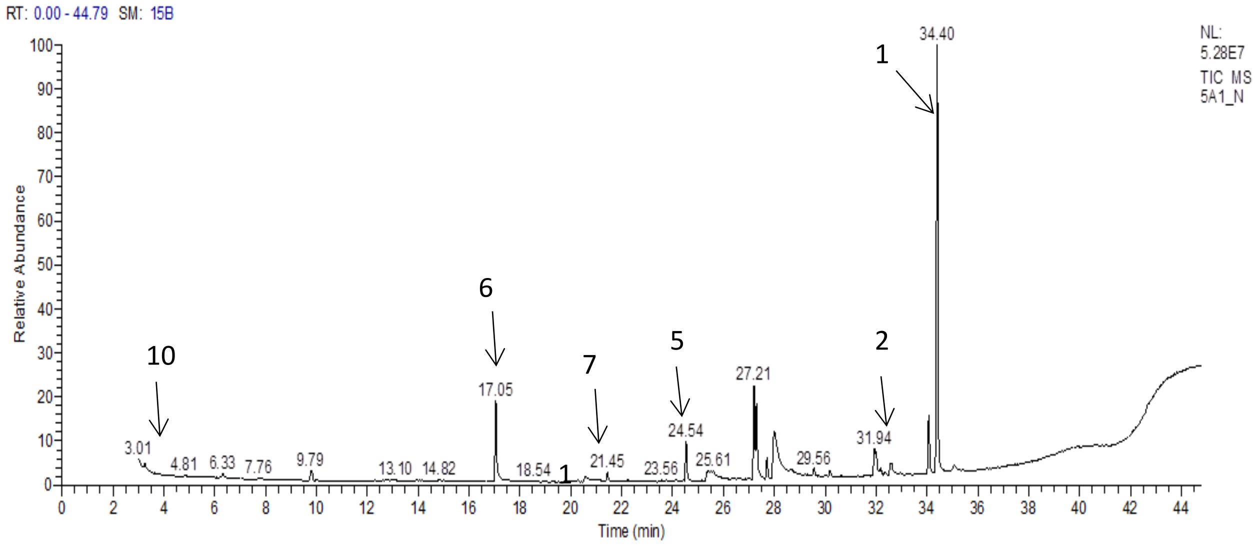

D

E

F

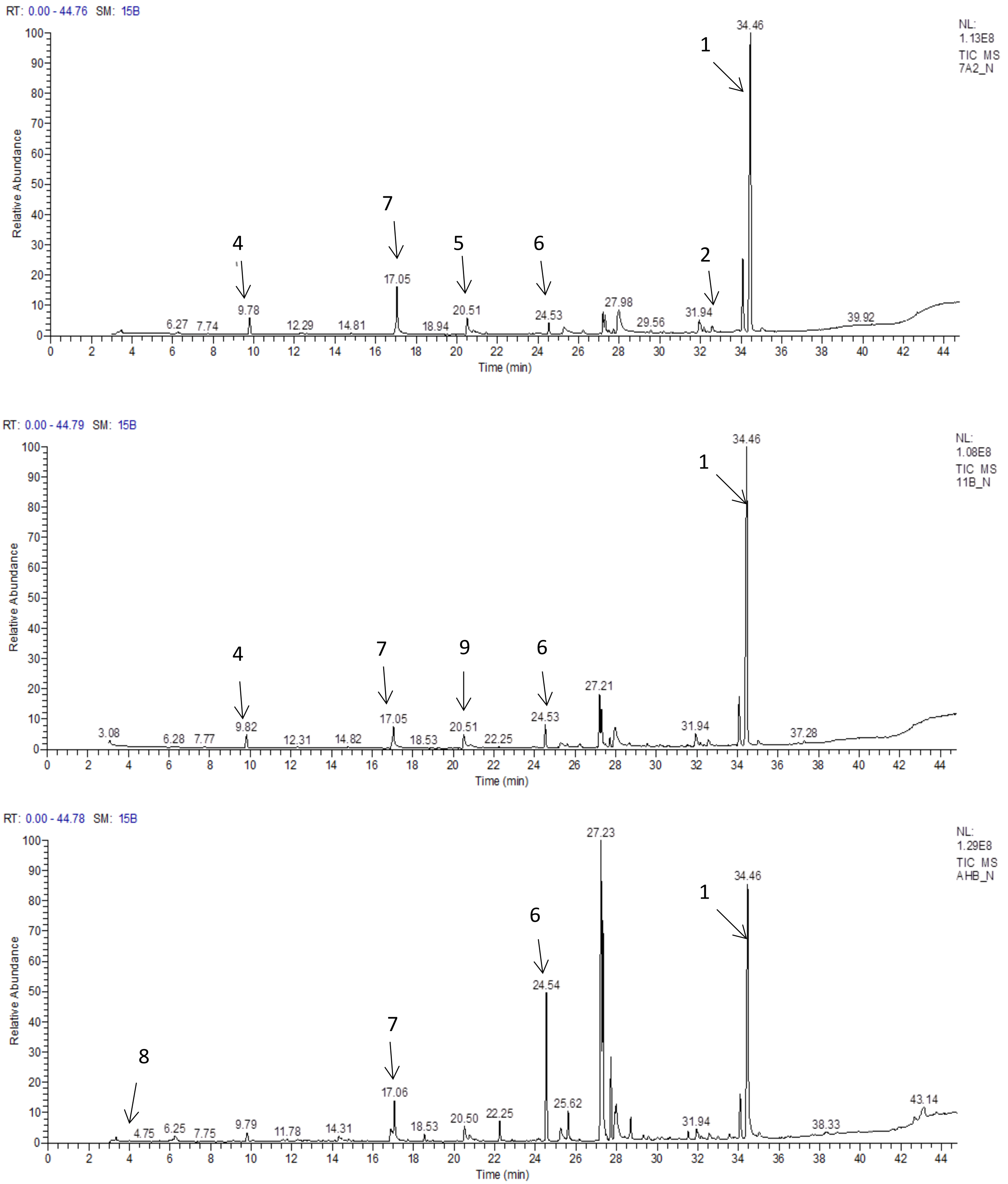

Figure S1. GC/MS profiles of MM supplemented with λ-Cyhalothrin after incubation for 24 h at 150 rpm and 37°C. A) GC/MS spectrum of control (non-inoculated MM+100mg/L λ-Cyhalothrin). B) GC/MS spectrum of MM+100mg/L λ-Cyhalothrin inoculated with 4B. C) GC/MS spectrum of MM+100mg/L λ-Cyhalothrin inoculated with 5A. D) GC/MS spectrum of MM+100mg/L λ-Cyhalothrin inoculated with 7A. E) GC/MS spectrum of MM+100mg/L λ-Cyhalothrin inoculated with 11B. F) GC/MS spectrum of MM+100mg/L λ-Cyhalothrin inoculated with AHB. The numbers refer to the compounds in table2.
